# Report on the Progress of Human Anatomy and Physiology in the Years 1844-5

**Published:** 1846-04

**Authors:** James Paget

**Affiliations:** Lecturer on General and Morbid Anatomy and Physiology, and Warden of the Collegiate Establishment, at St. Bartholomew's Hospital


PART FOURTH.

### Original Reports and Memoirs.

### REPORT ON THE PROGRESS OF HUMAN ANATOMY AND PHYSIOLOGY IN THE YEARS 1844-5.

By James Paget,

Lecturer on General and Morbid Anatomy and Physiology, and Warden of the Collegiate Establishment, at St. Bartholomew's Hospital.

The following Report concerns the history of physiology during a longer period than either of the two former Reports, and contains notices of the works published between the 1st of October 1844, and the 31st of December 1845. Its general plan is similar to that of those already published, except in that, for brevity's sake and for convenience of reference, there are appended to each chief section the titles of such essays relating to the subject therein treated of as, for various reasons, could not or needed not to receive any further notice. For this, as for all the former Reports, the original essays have been read in nearly every instance; and whenever reference is made in the foot-notes to more than one publication as containing the facts to which any part of the Report relates, the first of the books so referred to is that from which the account given in the text was derived.

### CHEMICAL COMPOSITION OF THE BODY.

*Elementary constituents.* Another fact in evidence that copper may occasionally exist, independent of poisoning, in the body, is furnished by Bertozzi,* who has detected it in fourteen biliary calculi of various kinds. The more yellow biliary colouring matter they contained the more abundant was the copper: white biliary calculi contained no trace of it. In bile he never could detect it. But, though these may be facts, yet the question, whether either copper or lead ever exists in the healthy body, must still be held open: in the last year MM. Devergie, Barse,† and others have again asserted that they do thus exist, and MM. Flandin and Danger‡ have as positively again denied it.

*Proteine compounds:—Albumen, &c.* M. Wurtz§ has rendered a further account of his mode of preparing albumen, in a state of purity, yet soluble without the addition of alkali or any other of the substances hitherto supposed necessary to its solution.

Dr. Ludwig|| has extracted from the materials hitherto vaguely named *Extractive matters* of the blood a principle which is isomeric with the binoxyde

* Oesterr. Med. Wochenschr. Sept. 6, 1845.
† Comptes Rendus, 28 Oct. 1844.
‡ Comptes Rendus, 30 Sept. 1844.
§ Annales de Chimie et de Physique, Nov. 1844.
|| Annalen der Chemie und Pharmacie, Oct. 1845.

of proteine of Mulder. The extractive is obtained by the pressing of defibrinated and coagulated blood, the neutralizing of the fluid pressed out, and again coagulating it, and filtering. To this filtered fluid, now free from albumen, alcohol is added, and the precipitate thus obtained, being washed with water, yields chloride of sodium, phosphate of soda, and a very small quantity of substance like binoxyde of proteine. Boiling alcohol removes fatty matters from it, and ether a crystallizing fat. The body thus purified, both in its general characters and in the results of its chemical analysis, resembles in all essential respects the binoxyde of proteine. It exists in the serum—not in the blood-corpuscles—and, next to the saline constituents, forms the chief part of the extractive matters of the blood of man, and of the dog, ox, sheep, and pig.

But what binoxyde of proteine may be is a question ; for doubts are now cast by Liebig* on the accuracy of Mulder's researches from which he deduced the existence of proteine itself. Liebig finds that the supposed binoyxde [tritoxyde?] of proteine obtained by adding ammonia to the solution of fibrine in dilute hydrochloric acid (the albuminose of M. Bouchardat), contains really all the sulphur which was present in the fibrine. And he finds that, when, as in the process proposed by Mulder, fibrine, albumen, or caseine is dissolved in potass ley, and the solution is neutralized by acetic acid, this solution contains no sulphuret of potassium or other sulphur compound; but that, if the precipitate formed when the acetic acid is added (and which is the supposed proteine) be dissolved in potass, sulphur may be detected in it by adding sugar of lead to the solution. This precipitate, therefore, Liebig holds, is not proteine; neither does he find the corresponding precipitate obtained from peas to be free from sulphur; nor, finally, has he "yet succeeded in producing a non-sulphuretted substance possessing the composition and properties of the so-styled proteine."

Dr. Buchanan† has found the remarkable fact, that the liquid of hydrocele may be coagulated in five or more minutes into a transparent tremulous jelly-like substance, by adding to it a small quantity of the washed clot of blood. The same coagulating property is possessed in a less degree by several other animal substances ; e. g. the buffy coat of blood in minute shreds, or even when dried and pulverized ; the transparent coagulum on a blistered surface ; and, in various much less degrees, by muscle, skin, cellular membrane, spinal marrow, mucus, and pus. He thinks that the coagulant power is chiefly seated in the colourless blood-corpuscles, which exist, together with the insoluble parts of the red corpuscles and some fibrine, in the washed clot; and with fibrine in the buffy coat; and in the coagulum from blisters. To these also he ascribes, I think, the usual coagulation of the blood ; the colourless corpuscles inducing the coagulation of the fibrine dissolved in the liquor sanguinis, and thus leaving its other constituents as serum, which is only *defibrinated* liquor sanguinis.

*Fibrine.* Dr. Polli‡ has confirmed the fact, that when fibrine begins to putrefy in water, albumen is among the substances formed from it. He has also found that if serum be mixed with three or four times its volume of water, and then boiled, the milky fluid which remains, and which contains, according to Boudet, albumen combined with soda, is spontaneously coagulable. If left to itself for a few days it forms an opaque-white soft clot, from which a

* Lancet, Feb. 20, 1845.

† Medical Gazette, Aug. 8, 1845. Dr. Buchanan argues as if it were proved that the fluid of hydrocele contains fibrine, and that it is fibrinous coagulation by which it becomes jelly-like when the portions of clot or other substances mentioned are added. It may be so, but there is no proof of it; and he is not right, I think, in saying that hydrocele fluid is commonly regarded as analogous to liquor sanguinis. The same coagulability may be seen in other dropsical fluids, e.g. when blood is let into them after death.

‡ Annali Univ. di Medicina, Feb. 1845.

limpid watery fluid separates, in which the clot sinks. But the spontaneous coagulation is the only circumstance in which it imitates fibrine, for the clot is not filamentous.

The rest of this paper is occupied with the changes induced in the fibrine of blood by inflammation, especially its diminished tendency to coagulation, and its rarefaction. Several cases of late coagulation are given, similar to that mentioned in the last Report,* but less remarkable than it. They confirm his belief that no blood is really incoagulable or remains liquid out of the body till it decomposes: and that when blood has seemed not coagulable, as in typhus and scurvy, its coagulation has been overlooked, or it has not been kept long enough for an unusually slow coagulation to take place. He considers that the most essential condition of this slowness of coagulation is a peculiar modification of the vitality of the fibrine, other favorable conditions being the excess of carbonic acid and of salts in the blood. And for fibrine thus modified by the influence of an inflammation he proposes the name of *bradyfibrine*.

Fibrine modified in another manner, and peculiarly *rarified*, he calls *parafibrine*. He has found that in the most acute inflammations the specific gravity of the blood or liquor sanguinis is rather increased by the removal of the fibrine; so that, contrary to the rule of health, the serum has a higher specific gravity than the liquor sanguinis. He supposes that this light fibrine, or parafibrine, may be that which is formed during, and as a result of, the inflammation, and which, in severe cases, may be formed in such quantity that it more than balances the effect of the ordinary fibrine and the *bradyfibrine* in increasing the specific gravity of the blood, so that the specific gravity of the blood is increased when both it and they are removed from it. He describes the parafibrine as coagulating very slowly into a mass which has a gelatinous aspect, and consists of very slender filaments, like those which give consistence to the albumen of an egg. When the serum is pressed from such a clot it becomes fibrous, tenacious, and heavier than the serum. Such clots are found in vesications : the fluid they contain is rich in parafibrine, and may be studied for the characters of this substance.

Both these modifications of fibrine may coexist in various proportions with ordinary fibrine in inflammatory blood, and hence arise many varieties in the time of coagulation and the appearance of the buffy coat of such blood. It is this light fibrine in the blood in inflammations, which, according to Dr. Polli, is effused with the serum in inflamed parts, for its tenuity permits it to traverse the walls of the vessels more easily than any other constituent of the blood can; and, once effused, it coagulates and becomes fibrous. Its copious existence in blood indicates the highest degree of inflammation; of this the gelatiniform buffy coat is the best sign. The simply retarded coagulation, due (at least in part) to the formation of bradyfibrine, indicates a lower degree of inflammation. The mere increase of ordinary fibrine is characteristic of the first degree of the same, and the increase of the one is proportionate to the extent of the other. Lastly, the existence of light fibrine in blood explains how a buffy coat may in certain stages of inflammations be formed in blood which coagulates quickly. In such a case the liquor sanguinis is very light, and may have been made lighter by previous bleedings ; the corpuscles sink rapidly and leave above a layer which may presently form the buffy coat.

*Saline constituents of animal fluids.* Dr. Golding Bird† questions the correctness of Enderlin's‡ deduction that no organic acid is combined with the alkaline bases of any of the secretions except the bile, because no alkaline carbonate is found in any of their ashes. He thus shows that a salt of soda

* Page 6. † Philosophical Magazine, May 1845. ‡ See last Report, p. 8.

with an organic acid may exist in a solution of phosphate of soda, and yet yield no carbonate on ignition;—nine grains of dry tribasic phosphate of soda being mixed with four grains of acetate of soda, and exposed to a full red heat in a covered crucible, the product is easily soluble in water, and has a strong alkaline reaction; but no effervescence ensues on adding dilute sulphuric acid to it.*

### BLOOD.

*Corpuscles.* Mr. Gulliver† has published a synopsis of all his former observations on the sizes of the red corpuscles of the vertebrata, in tables, stating the measurements of these bodies in no less than 485 species. He has also shown,‡ in reference to the formation of the buffy coat, that the rapidity of sinking of the blood corpuscles is not directly proportionate to the tenuity of the fluid in which they are collected, but rather, inversely proportionate; for it depends mainly on the rapidity and completeness with which they aggregate in rolls and clusters (as described by Hewson and others). This grouping is promoted by saline solutions, which have been made thicker by mixing gummy matters with them, and is retarded or destroyed by dilute saline solutions. As soon as the corpuscles are aggregated they begin to sink rapidly; and hence, in blood that will be buffed, there is a remarkable acceleration of the sinking of the corpuscles two or three minutes after it is drawn.

*Blood.* Dr. G. O. Rees,§ extending his well-known observations on the changes produced in the blood corpuscles by the entrance of fluid when they are placed in fluids of less specific gravity than the liquor sanguinis, and by the exit of fluid from them when placed in fluids of higher specific gravity than their fluid contents, has shown in these changes several sources of fallacy in the quantitative analyses of the blood. He states also that, after copious perspiration induced by exercise, the corpuscles are thin and very like those from which fluid has exuded when immersed in a denser fluid. The change may be ascribed to the loss of water from the liquor sanguinis, and the consequent increase of its density, till it becomes of higher specific gravity than the fluid contents of the blood-corpuscles. On the other hand, the serum of a dog, into whose veins six ounces of water were injected in the place of six ounces of blood just previously drawn, was tinged by the colouring matter of the corpuscles, which had oozed from them into the fluid of reduced specific gravity.‖ In examining corpuscles retained at a temperature of 100° F., he has seen each corpuscle contracting into a hour-glass form, and then dividing into two, usually of unequal size, a process which he supposes to be similar to that by which the corpuscles are naturally multiplied.

*Gaseous contents.* Prof. Magnus¶ has repeated his experiments to determine the quantities of oxygen and nitrogen contained in the blood, and the extreme quantities of those gases which it is capable of absorbing. The ex-

* Among other works relating to animal chemistry in general, the most important are, of course, the Lectures of Liebig in the Lancet of the past year, and the continuation of Mulder's Physiol. Scheikunde; and after these,—1. An essay by Dr. Schmidt, 'Zur vergleichende Physiologie der wirbellosen Thiere,' in the Annalen der Chemie, Juni 1845; from an essay published at Brunswick. Its main purpose is to obliterate the supposed line of absolute material distinction between the animal and vegetable kingdoms, evidence being drawn from ample analyses of the elementary tissues of the invertebrata. 2. Gobley, "Sur l'existence des acides oléique, margarique, et phospho-glycérique dans le jaune d'œuf," in the Comptes Rendus, Sept. 29, and Nov. 3, 1845. I may here also refer for the compendium of every recent work on chemistry to the Annuaire de Chimie of MM. Millon and Reiset. Paris, 1846.

† Proceedings of the Zoological Society, No. clii, Oct. 14, 1845.

‡ Dublin Medical Press, Dec. 11, 1844, and Edinb. Med. and Surg. Journ., Oct. 1845.

§ Gulstonian Lectures. Medical Gazette, March 14, e. s. 1845.

‖ Schultze has made a similar observation in blood drawn after taking large draughts of water.

¶ Philosophical Mag., Dec. 1845, Suppl. p. 563, from the Annalen der Physik u. Chemie, lxvi, p. 177.

periments consisted chiefly in repeatedly agitating fresh drawn blood with atmospheric air, and then (with necessary cautions) washing out the absorbed oxygen and nitrogen with carbonic acid. The results of numerous experiments were pretty uniform. The quantity of oxygen obtained from the blood was from 10 to 12·5 per cent. of its volume, that of nitrogen from 1·7 to 3·3 per cent. Reversing the mode of experiment, it was found that blood could absorb 1½ times its volume of carbonic acid, and that, after all its oxygen and nitrogen had been washed out by carbonic acid, it could absorb, at a maximum, 16 per cent. of its volume of oxygen, and 6·5 of nitrogen. Similar experiments were made on old horses' arterial blood, and they proved that such blood naturally contains in simple solution from 10 to 10·5 per cent. of oxygen, and from 2 to 3·3 per cent. of nitrogen. And some further experiments lead the author to believe, that of this 10 per cent. of oxygen in arterial blood, about 5 per cent. are consumed in the systemic capillaries, and 5 per cent. remain in the venous blood, to be completed to the general average of 10 per cent. by the absorption of fresh oxygen in the pulmonary capillaries.

*Life: capacity of organizing.* Whatever doubt might still remain in the minds of some, whether Mr. Hunter maintained the truth in his doctrine that the blood could be organized, that is, that the whole substance of a portion of blood, being at rest either within or without a vessel, may develope itself into tissue and become vascular, and grow and nourish itself, must be satisfied by the observations of Dr. Zwicky* on the metamorphoses of the thrombus, or clot of blood which forms in a vessel above a part at which it is tied. For observations which, like these, stand on the boundary between physiology and pathology, no more space can be given than to state briefly the result; which is, that, to all microscopic appearance, the organization of the fibrine in an inflammatory condition and of that in a clot of blood is in all essential respects the same process, the presence of the blood corpuscles in the latter having no further influence on the process than that of somewhat retarding it. The whole process of the metamorphosis of the fibrine of the blood into fibro-cellular tissue, and of the formation of vessels in it is satisfactorily traced; and thus the only additional evidence which was needed is supplied for the establishment of another of the long-doubted Hunterian doctrines.

*Modifications in disease.* An account has already been given of the researches of Dr. Polli on the changes of blood in inflammation. The continued investigations of MM. Andral and Gavarret† have in numerous striking instances confirmed their account of the constant increase of fibrine in the blood in direct proportion to the increasing acuteness of the disease in all cases of inflammation, and of its regular and proportionate decrease in typhoid fever and purpura.

And these cases are confirmed by the laborious investigations of MM. Becquerel and Rodier,‡ who have prefaced their pathological results by certain facts of more present interest in physiology. They give a standard table of the comparative constitution of the healthy blood of the adult male and female, showing the constant differences between them. As to the differences of constitution of the blood at different ages, they find none that is well-marked in men, but in women it is constant that the proportion of corpuscles is less before menstruation is fairly established than it is afterwards, and that when menstruation has ceased it again diminishes. In both sexes the cholestearine increases with increasing years after the age of between forty and fifty. The corpuscles are more abundant in the robust and well-fed. During pregnancy there is a great diminution in the proportion of corpuscles, a less considerable one in that of albumen, a slight increase of fibrine and of the

* Die Metamorphose des Thrombus; von Dr. H. Zwicky. Zurich, 1845. 4to.

† Comptes Rendus, Séance du 18 Nov. 1844. ‡ Ibid. and Gazette Méd., 23 Nov. e. s.

phosphorized fatty matter, and an increase in the proportion of water. The other results obtained by these authors relate more exclusively to pathological states of the blood, but they are in that regard of very high interest.*

### GENERAL ANATOMY AND PHYSIOLOGY OF THE TISSUES.

*Fibro-cellular tissue and elastic tissue.* Distinct filaments are described by Stadelmann† as demonstrable in the transverse sections of fibro-cellular tissue, cut and moistened after being dried. Their cut ends are like exactly circumscribed, pellucid circles, shadowed by sharp lines. Their diameters are all equal, and, as nearly as they could be measured, $\frac{1}{19000}$ of an inch. After similar preparation of portions of the elastic tissue, he finds the cut ends of its fibres round, ovate, triangular, or polygonal, circumscribed by pellucid lines, and separated by intercellular substance. They measure in the human ligamenta subflava from $\frac{1}{9174}$ to $\frac{1}{7037}$ of an inch: in the ligamentum nuchæ of the horse they are nearly twice as large.

*Membranes.* Much attention has been given to the arrangement of nerves in the serous membranes. The republished observations of Purkinje,‡ on the nerves of the pia mater and arachnoid are noticed elsewhere. They are confirmed and added to by those of Mr. Rainey,§ on the nerves in the arachnoid; but these have been published since the time to which the Report has reference. M. Bourgery|| also has given a long account of what he believes to be nerves of the peritoneum, dissected by the help of weak nitric acid, and briefer notices of those of other serous membranes; but, in regard to the nerves of the peritoneum, since the cords which he dissected were not submitted to minute microscopic examination, and the microscope can discern only obscure traces of any nerves at all in that serous membrane, his description cannot yet be received as sure. So also, it must remain an open question, what amount of nerves the pleura receives, and how they are arranged ¶

*Cuticles, pigment, &c.* E. H. Weber** has noticed that in all cases, but especially in the nasal ciliary epithelium of warm-blooded animals, the ciliary movement is accelerated by warmth and reduced to half its usual rate by cold. In this as well as in its rythmic action the ciliary movements are like those of the heart; and there is no striking difference between them in regard of their period of continuance after apparent death, for he has seen the frog's auricle contracting for sixty hours, and after the blood in which it was placed had become quite putrid.

Dr. Moleschott†† has examined the discoloration of the pigment of the

* Papers on the development of the blood corpuscles have been read to the Royal Society by Mr. Newport and Mr. Wharton Jones, but I cannot gather an intelligible account of their results from the abstract given in the Proceedings of the Society, Nos. 60 and 61.

There is an agreeably written sketch of the present state of the pathology of the blood, by Wunderlich, with the title 'Pathologische Physiologie des Blutes, Stuttgart, 1845,' but there is nothing in it, I think, which is both new and interesting in physiology. Here also, as well as for the anatomy of the tissues, I may refer to Prof. Harting's 'Recherches micrométriques,' 4to, Utrecht, 1845, containing exact measurements of the blood corpuscles and other parts. The physiological part of the work is preceded by a careful discussion of the comparative merits of different methods of micrometry, among which, after minutely testing all, the author prefers the common plan by double sight, because it is the most convenient, always applicable, and, for very minute objects, liable to least error.

† Sectiones transversæ partium elementarium corporis humani; Turici, 8vo, 1844, pp. 10 and 12.

‡ Müller's Archiv, Hefte iii, iv, 1845.

§ Report from the Med. Chir. Society in the Lancet, &c., February 27, 1846.

|| Comptes Rendus, 8 Sept. and Gazette Médicale, 20 Sept. 1845.

¶ See hereon Pappenheim, in the Comptes Rendus, Oct. 1845, and Purkinje l. c. p. 293.

** Archives d'Anat. génér. et de Physiologie, Janvier 1846, from a paper read at the Scientific Congress at Naples in 1845.

†† Tijdschrift voor Naturl. Geschiedenis en Physiologie, 1845, St. 2, D. 12.

skin of frogs when they breathe in pure oxygen, held over water, so that the carbonic acid they produce may be absorbed. In a few hours the blackish-green elongated marks, extending backwards and downwards from the eyes, become lighter and greener. Then for several days the change goes on more slowly, but at the end of twelve days it is very distinct in all the spots; the dark semicircular marks on the thighs can then hardly be seen.

The development of epidermis has been described by Mr. Erasmus Wilson* as consisting essentially in—1st, the production of "primitive granules" in the blastema; 2d, the collection of four, five, or six of these into "aggregated granules," which become the nucleoli of the complete cells; 3d, in the arrangement of a single tier of aggregated granules around each of these first aggregated granules, so as to form an oval or circular mass, a "nucleated granule," the nucleus of the complete cell; and 4th, the production of another tier of aggregated granules around the nucleated granule, forming a transparent border around it, around which border there is probably a cell-membrane, forming the proper epidermis cell or "nucleolo-nucleated cell." The growth of the cell he believes to be due to successive repetitions of the same process in the development and growth of aggregated granules within it, the full-grown cell containing secondary and tertiary cells and bodies in all the four stages of development above mentioned.

*Cartilage.* M. Valenciennes† has made an extensive examination of the structure of the cartilages in mollusca and cartilaginous fish, but the only facts which it appears important to state here are—1st, that the cartilage cells are generally arranged in such regular plans that it would be possible to determine by microscopic examination the order, or even the genus, of an animal, from the character of its cartilages; 2d, that none of the cartilage cells have, in any species, canaliculi communicating with them; and 3d, that gelatine, not chondrine, is abundant in the cartilages of cephalopods.

*Bones: chemical analysis.* A number of analyses of bones of various animals have been made by Dr. Stark.‡ He shows that, besides their marrow [i. e. I suppose, all the fat that can be easily scooped out of the cancellous tissue], the bones of mammalia contain from 13·5 to 29·2 per cent. of fatty matter. The solid part of the shaft of the canon bone of a sheep contained 4·3 per cent. The bones of almost all birds under one year old contain an oleo-albuminous (?) matter, which, as the bird grows older, is absorbed. No less than 232 analyses of various bones from all the vertebrate classes are given, to show the proportions of animal and earthy matters; and the result is, that in all bones the proportion is nearly the same, the average proportion for all being 66·09 of earthy matter to 33·91 of cartilage. No confirmation is afforded of the notion that the proportion of earthy matter is the larger the higher the animal is in the scale of creation, for the largest proportion (68·74) was in the true bones of cartilaginous fishes—the smallest (61·9 to 62·3) in the bones of the bear and marmozette monkey. The proportion of earthy matter appeared a little greater in the wild than in the domesticated mammalia. There was no evidence found of its increasing with age in either men, cows, or sheep. Neither did the hardness, or inflexibility, or want of transparency of bones appear to be dependent on the earthy matter being present in a very large proportion, but on the mode in which the textures of the bone were held together.

*Structure.* Mr. Goodsir§ states that in the cavity of each bone-corpuscle, but not extending into the canals, there is "a little mass of nucleated cells of great transparency." This he regards as the *germinal centre* of the texture or

* Proceedings of the Royal Society, No. 61, June 19, 1845; and Philosophical Mag., Feb. 1846.
† Comptes Rendus, 25 Nov. 1844. ‡ Edinb. Med. and Surg. Journ., April, 1845.
§ Anatomical and Pathological Observations. Edinburgh, 1845. 8vo, No. x, p. 64.

of the cells comprehended within the canals of the corpuscle which it occupies, through which canals, also, he supposes that the nutritive material which the mass of cells attracts into the corpuscle is conveyed for the nutrition of the hard parts between them. He also describes the tissue which lies between the blood-vessels and the walls of the Haversian canals as a layer of cellular substance, at least in the fœtus, for in the adult it is replaced by fat.* In another paper† he describes, (as a source of fallacy in those experiments in which it appeared that, when a piece of bone was removed and its periosteum was left, the periosteum reproduced the lost bone,) how that it is hardly possible to separate periosteum from bone without detaching minute portions from the surface of the bone; and that these, adhering to the periosteum, may serve as nuclei for the production of the new bone, which thus seems to be produced from the periosteum itself.

*Development.* The early stages in the development of bone are described by Köstlin,‡ from the examination of a thin layer of new bone (osteophyte), which lined nearly the whole skull of a woman who died three weeks after delivery. His account has, perhaps, less of special than of general interest, for it may not be true of the ordinary development of bones, but it confirms the account given by Karsten§ and some others, of the general plan of cell-development. He finds that, in a homogenous membrane formed in the earliest stage of the exudation, there are first formed scattered elementary granules. These gradually increase in number; some enlarge, some coalesce, and the larger granules thus formed separate into a moderately thick wall, a dark contents, and an eccentric nucleus. Thus they become primary cells; and these also enlarge by growth and coalescence. At first their form is nearly spherical, but they gradually become less regular, and, their contents disappearing, they become also less turgid and clearer, and their nuclei more numerous. As their contents disappear so their walls coalesce with the intercellular substance, and their nuclei become imbedded in it. The number of these nuclei increases still more and they become darker by the in-taking of earthy matter; and, moreover, there is formed from the intercellular substance around each cell a group of dark corpuscles like the nuclei, which acquire a regular arrangement in regard both to them and to one another. Their arrangement is such, that if one imagines them connected with one another and with the nuclei by dark lines, and the cell spaces darkened by the reception of earthy matter, one will have the common bone-corpuscles with the dark network of the so-called calcigerous canals.

*Periosteum.* Purkinje‖ has found (as Pappenheim¶ also did) a copious network of nerve filaments in periosteum. He examined particularly that of the front of the tibia, and that of the vertebral canal. In the latter, which he describes as formed by an outer layer of the dura mater, divided into two layers at the foramen magnum, he found abundant bundles of fine-fibred nerves, which appeared to communicate through the intervertebral foramina with the sympathetic.

*Muscular tissue: its structure, action, and growth.* Stadelmann,** in transverse sections, could never detect any appearance of an empty space or canal in the axis of the striated muscular fibre, such as Valentin and others have described.

E. Weber†† has examined the mode of contraction of the muscular fibres

* I cannot make out what is described as the mode of origin and development of the cells of this layer of substance.

‡ Müller's Archiv, 1845, Heft. i.

§ See last Report, p. 37.

‖ Müller's Archiv, Heft. iv, p. 289.

¶ Last Report, p. 10.

** Archives d'Anat. Gén. et de Physiologie, Janvier 1846, from the Report of the Scientific Meeting at Naples in 1845.

†† L. c., p. 15. The rest of his account of this tissue confirms Mr. Bowman's.

under the favorable condition of continued contraction, into which the frog's muscles are thrown when excited by the electric current from a rotating magnet. He finds that the contraction is always by simple shortening, and never by zig-zag flexures of the fibres; and that these are formed, as Mr. Bowman stated, only when the fibres relax, and by their elasticity tend to elongate. He shows also that, in contraction, the fibres do not become harder, and that it is only their increased tension when contracted which makes them feel harder, as it does also the tendons and other tissues at the same time.

Dr. Helmholtz* appears to have obtained a more direct proof than hitherto existed, that chemical decomposition accompanies the action of a muscle. With all due precaution he electrified the hind legs (say the right hind legs) of frogs till their muscular irritability was exhausted, and then compared the results of the analyses of the muscles of these and of the other hind legs of the same frogs, which, except in that they had not been electrified nor in any way excited to muscular action, had been kept in the same external conditions. The result was, that in every case the quantity of alcoholic extractive matter was found to be increased in the electrified muscles, and the quantity of water-extractive matter decreased in them. The proportionate quantities of the former in the electrified and in the non-electrified were (on an average of six experiments) as 1·33:1, and those of the latter as 0·78:1. Similar results were obtained by like experiments with an eel-pout and with a pigeon. Of the other constituents of the muscles;—the changes, if any, in the fibrine could not be determined; the differences in the quantity of albumen were inconsiderable; the fat appeared unchanged. Counter-experiments were made, which showed that electricity did not produce similar changes in muscles whose irritability had been previously exhausted, and that the changes were not due to ordinary spontaneous decomposition.

Together with many observations on the minute structure of the muscles of hymenoptera, Prof. Harting† has given an account of the comparative dimensions of the muscular fibres of the new-born child and adult, showing that the average diameter of the primitive fibres in the child is to that in the adult as 1:3·64, and the respective average intervals between the transverse striæ as 1:1·18. In the child the distance between the striæ is to the width of the fibre as 1:4·415; in the adult as 1:8·42. It is hence deducible that the ordinary growth of a muscle is due to the increase in size, not in number, of its primitive fibres; but whether this increase of size is due to enlargement, or to increased number, of the primitive filaments, could not be determined. It is evident also, that of the growth of a muscle in its length, part is effected by the increase in the length or depth of the particles (?) on which its transverse striation depends; but this is not enough to account for the whole growth of a muscle between childhood and manhood; so that part of the growth in length must be ascribed to the increased number of these particles (?).

He has also measured the muscles and their elementary parts in the healthy and the paralytic arm of an adult; from which it appears that the diminution of size accompanying the paralysis of the muscles was due to a diminution in the size, not in the number, of the primitive fibres. But with the diminution in the size of the muscles there was no corresponding diminution of the nerves: both their trunks and their ultimate fibres were of equal size in both arms.‡

* Müller's Archiv, 1845, Hefte i, ii. † Tijdschr. voor Naturl. Geschied. en Physiol. d. xii.

‡ See further on this subject in the section upon Nutrition. And on the several tissues refer to. 1. C. Bruch, Untersuchungen zur Kenntniss des körnigen Pigments; Zurich, 1844, 4to, containing a clear account of all that is known of pigments, whether healthy or morbid, with some original observations. 2. J. E. F. Knorz, De pili structura et genesi; Marburg, 1844, 8vo, of which a similar account may be reported. 3. Dr. Gregory, On the presence of Phosphate of Magnesia in Bone, and on a new method of obtaining Phosphoric Acid from Bones; in the Medical Gazette, April 11, 1845. 4, C. G. L. Bruch, Nonnulla de Rigore Mortis; Magunt, 1845, 4to, confirming by new evidence the

CIRCULATION.

*Action of the heart.* Dr. Mitchell,* in a case of ectopia cordis, watched the movements of the heart for an hour and fifty minutes. The pulsations were twenty-five in a minute before the separation of the umbilical cord; after it they fell to twenty, and then to seventeen. After the auricles were distended with blood they emptied themselves by a gentle flowing motion, and immediately after this the ventricles contracted. The effect of the ventricular contraction was to shorten the heart from base to apex, and to cause a considerable bulge or projection in the centre, giving rise to an evident elevation of the fingers when laid on it. The apex of the heart was *not* elevated. After the ventricular contraction the heart appeared quite flaccid and relaxed, although it was evident that the ventricles were not emptied.

Volkmann† has discussed the relation of the movements of the heart to the nervous system; and, to prove that they do not depend on the brain and spinal cord, he adduces the fact that they continue after the heart is cut out, while all the rythmical movements which do depend on the brain and cord, such as those of respiration and the lymph-hearts, cease as soon as their connexion with parts of those nervous centres is destroyed. He supposes, therefore, that the movements of the heart depend on the *immanent* power of the sympathetic nerve-fibres and ganglia in its substance. His experiments show, that if the auricle and ventricle, while pulsating rythmically and in harmony in a fresh frog's heart, be suddenly separated from each other, though they may both continue to pulsate they will not pulsate in harmony. And when the ventricle is divided by incisions carried through nearly its whole length, some of its portions will continue to pulsate spontaneously and rythmically, while others, just as large, will only move when irritated. He thinks that this shows that, in the former, central organs remained from which impulses for movement might proceed, while in the latter there were no such central organs or ganglia. He concludes, therefore, that the ganglia in the heart are so many central organs, or points from which motor impulses flow out, and that they are suited for action in concert by connecting nerve fibres, forming altogether a system so arranged as to produce, in regular series, the successive contractions of the muscles of the heart.

This theory coincides in many points with that of Kurschner, alluded to in the last report; and he holds that the auricles are the parts to which the reflex influences of the ganglia are always first directed, and that the contraction of the ventricles is the consequence of the contraction of these. In evidence of this he says, that whatever part of a heart, when cut out but still irritable, is irritated, the consequent contraction always begins in the auricle. [But it is certainly not always so: I have many times tried the experiment in cut out turtles' hearts, and have always found that the contraction begins at the part irritated and thence extends over all the rest.] Valentin,‡ also, has made experiments on the same question, but he doubts whether the rythmical contractions of the heart are dependent wholly on its nervous system, and not in part on the mechanical arrangement of its fibres; since, he says, the continuance of rythmical movements is observed only in those pieces of the heart established view that the rigor is due to the contraction of the muscles. 5. Harting, Histologische Aaanteekeningen; in the Tijdschrift already referred to. Besides the papers on the muscles, lens, and nerves, of which an account is here given, the essay contains remarks on the milk-corpuscles, the action of sublimate on the blood-corpuscles, and other questions of the anatomy of tissues. The continuations of the admirable works of Dr. Sharpey, in the new edition of Quain's Anatomy, of Dr. Todd and Mr. Bowman, in their Physiological Anatomy, and of Mulder, in his Physiol. Scheikunde, are also important contributions to the recent history of general anatomy.

* Dublin Journal of Med. Sciences, Nov. 1844. † Müller's Archiv, 1844, Heft iv, p. 424.

‡ Handb. der Physiologie, Bd. ii, p. 767.

which remain connected with portions of the tissue intermediate between the auricle and ventricle. Kölliker,* also, while he agrees with Volkmann's explanation of the rythmical action of the heart, states this as a fact which he, like Valentin, has observed in frogs' hearts. [But in the hearts of turtles, the size of which makes the fact evident enough, it is very different; I have often seen the apex of the heart continuing its rythmical contractions long after being cut off from the rest of the ventricle. Both auricles also continue similarly contracting when cut off above the auriculo-ventricular rings; but their contractions are not synchronous. Indeed, I do not remember to have ever seen any difference, in respect of the continuance of their rythmical action, whether the pieces cut from the heart were or were not connected with the tissue uniting the auricles and ventricles.† I believe, therefore, that Volkmann's theory may be held as true; but that there is no evidence at present for assigning a peculiar locality for the chief centres for harmonizing the rythmic actions of the heart.]

*Arteries.* An account of the structure of the blood-vessels, confirming in all respects that of Henle, and supporting the microscopic by chemical evidence, so far at least as the action of acetic acid and potash on the several coats is concerned, is given by Mulder,‡ from examinations by himself and Donders.

The nerves of the arteries are among those which Purkinje§ has submitted to renewed investigation. He finds, by the help of acetic acid, all the plexus of arteries in the rete mirabile of the ox surrounded by a close web of nerves.

In an excellent essay on the anatomy of the parts concerned in laryngotomy and tracheotomy, Dr. Gruber|| has treated especially of the middle thyroid (A. thyroidea ima. seu Neubaueri) and the crico-thyroid arteries. The former occurs in about ten per cent. of the bodies examined, arising in general from the innominata, more rarely from the arch of the aorta or the common carotid, more rarely still from the inferior thyroid or thyroid axis, and most rarely from the internal mammary. It probably never arises from the left carotid; when it appears to do so the artery thus arising should be called a second inferior thyroid. Its diameter varies from half a line to two lines and a half; in two cases, with healthy thyroid glands, its trunk measured five lines. It is more frequently on the right than on the left side; and once only in ten cases was double. It may proceed vertically up the front of the trachea to the isthmus or either lobe of the thyroid gland, or it may pass obliquely across to the trachea, even going from its right side to the left lobe of the gland.

In regard to the crico-thyroid artery, Dr. Gruber notices especially that arrangement in which it is very large on one side, having a diameter of from three-quarters of a line to two lines. This is most often observed when there is a middle lobe to the thyroid gland, but the large artery is not always on the same side as the middle lobe. Such an artery occurs about once in every four persons, and is more frequent on the right than on the left side. Its place may be taken by the thyroid branch of the right superior thyroid artery. The large artery in most cases bends down at a right angle in front of the crico-thyroid membrane, to reach the isthmus of the thyroid gland, and gives off at its bend a small branch which crosses the membrane. More rarely the

* Die Selbständigkeit und Abhängigkeit des sympathischen Nervensystems. Zurich, 1844. p. 36.

† It is, however, I think, necessary for the propagation of contraction from one part of the heart to another, that they should be connected together by muscular tissue; probably because there are nerves in it. If two portions of a heart be held together by a very narrow isthmus of muscle, the contraction excited by irritating one will extend to the other; but this will not happen if the connecting isthmus be tendinous tissue, even though it be a piece of the auriculo-ventricular ring.

‡ Physiologische Scheikunde, St. vii, p. 664.

§ Müller's Archiv, Hefte iii, iv.

|| Oesterreich. Medic. Jahrbucher, Mai u. Juni, 1845.

trunk of the artery crosses the crico-thyroid membrane and reaches the opposite side. It gives branches to the larynx through the crico-thyroid membrane, to the middle lobe of the thyroid gland, to the lobe of its own side, and when it crosses over, to that of the other side also, and very often to the isthmus of the gland.*

*Capillaries.* Observations on the development of the capillaries in tadpoles and the tails of youug tritons by Platner† indicate, as some others have done, that new capillaries are formed by outgrowths from old ones. First, capillaries are seen which have abrupt blunt closed ends; some of these have no trace of prolongation, but many exhibit a very thin, long *outrunner* which is gradually lost sight of; and in some cases it may be seen how two such thin canals as these unite and form one arch, which gradually increasing becomes a new capillary loop. At first this is filled by finely granular matter, and is too small to admit blood-corpuscles. It soon acquires distinct walls, but the nuclei found on fully developed capillaries are subsequent formations.

*Veins.* Henle described generally the differences of structure of veins in different parts of the body according to the degree in which the longitudinally fibrous coat is developed; that in some it is hardly discernible, in others strongly marked; but he did not indicate the veins thus differently conditioned. The result of many dissections by Dr. Norman Chevers‡ is that, as a rule subject to but few exceptions, the deep-seated veins of the trunk have their proper or middle coat (wherein I suppose he includes all between the striated coat and the external cellular coat) composed almost entirely of circular fibres; while in the external and superficial veins there is in the same situation a strong internal layer of longitudinal fibres, and a thinner layer of circular fibres next external to it.

*Venous pulse.* Two cases of pulsations in the veins of the back of the hand, coincident with the pulse of the arteries, are recorded by M. Martin-Solon.§ Both occurred in patients who had been largely and repeatedly bled for pleuro-pneumonia. M. Poiseuille, in a discussion on the essays, referred to cases recently recorded in a thesis by Condret, in which the persons presenting the venous pulse were strong young men with full arterial pulses.||

RESPIRATION.

*Structure of the lungs.* Some interesting points in the anatomy of the lungs are determined by the investigations of Mr. Rainey.¶ The airpassages he divides

* For the practical deductions from these anatomical facts I must refer to the original paper.

† Müller's Archiv, 1844, Heft v.

‡ Medical Gazette, Aug. 8, 1845.

§ Bull. de l'Académie de Médecine, Nov. 1844, pp. 102-116.

|| All the physics of the circulation are well discussed by Bergmann in his article, "Kreislauf des Blutes," in Wagner's Handworterb. der Physiologie. There is an exposition of the theory of the wave-movement of the blood in the arteries, and of the mechanism of the pulse, by Dr. H. Frey, in Müller's Archiv, 1845, H. ii, iii. His essay is in full support of the theory as commonly received: against it there are, an anonymous very clever essay "On the Cause of the Pulse," in the Medical Gazette, July 11, 1845, and one with the same title by Dr. Thomas Williams, in the same, July 25, 1845. The opinions in these two essays differ in degree rather than in kind. It seems to me that the existence of what may be justly termed a wave of blood raised by each contraction of the ventricle, is quite certain: the main question is, how far does the first wave extend? None of the essays answer this question; and it is not possible to give an answer which shall be always true, for both the breadth and height of the wave must, in different cases, depend on very many variable conditions, of each of which we can as yet obtain only an uncertain estimate. Other works of more or less interest in the anatomy and physiology of the circulatory system are,—Parchappe, Sur la Structure . . . du Cœur; Paris, 8vo, 1844. Pliny Earle, Observations [with extensive statistics] on the pulse of the Insane; in the American Journ. of Med. Science, January 1845. Norman Chevers, On the effects of obliteration of the Carotid Arteries; in the Medical Gazette, Oct. 31, 1845. Bouisson, Sur les lésions des artères fessière et ischiatique; in the Gazette Médicale, Mars 1845, containing measurements of the exact relative positions of these vessels. Prevost et Lebert, Sur le développement des organes de la circulation . . . du poulet; in the Annales des Sc. Nat., t. i, 1845.

¶ Medico-Chirurgical Transactions, vol. xxviii, p. 581, 1845.

into the bronchial tubes and the intercellular passages continued from them, taking for the line of demarcation between them the parts at which the mucous membrane and ciliary epithelium of the bronchi terminate. These terminate abruptly in bronchi of from $\frac{1}{50}$ to $\frac{1}{30}$ of an inch in diameter; the mucous membrane retaining to this boundary its longitudinally and circularly fibrous character and its pale colour. Beyond the boundary, the tubular form of the air passages continued from the bronchi is for some distance retained, but it is more and more lost as the passages branch and approach the surface of a lobule, and as the number of air-cells which open into them continually increases. Thus, at last, each minute division of the air passages becomes quite irregular in its form, air-cells opening into every part of it, and almost constituting its walls, till itself ends, without dilatation, in an air-cell. Beyond the boundary at which the bronchial mucous membrane abruptly terminates the walls of the air-passages are formed only by a membrane similar to that which walls-in the air-cells; and they have no epithelium. Those air-cells are smallest and most vascular which are situated nearest to the centre of the lung; their size increases and their vascularity diminishes at the parts nearer the circumference—in adaptation, probably, to the larger and more ready supply of fresh air to the former parts. Those cells which open directly into the bronchial tubes and intercellular passages open into them by large circular apertures; they are themselves similarly opened into by other cells, which again are similarly opened into by others; forming thus, for each opening into a tube or passage, a series of communicating cells; each series reaching usually from the passage to the surface of the lobule. The cells which are placed in the angles of the branching of an intercellular passage probably open into both the branches, as well as into one another. The walls of the air-cells, on which the capillaries are placed, are formed by thin and transparent but fibrous membrane, which in the formation of the successive communicating cells is folded, or as if constricted, so as to form a definite sharp-edged circular opening of communication between each two, and between the first of the series and the passage into which it opens. In the reptiles the corresponding folds bordering the sacculi of their lungs inclose each a double or folded layer of capillary plexus; in the mammal's lung each such fold incloses a single layer of capillaries, so that both sides of all these capillaries are exposed to the air in the adjacent cells. And this plan of arrangement and structure of the air-cells exists in the mature mammalian fœtus just as it does in after-life.*

*Nerves of the lungs.* In his admirable essay on the physiology of the nerves, Volkman,† to prove the influence of the pneumogastric nerves on the bronchial tubes, relates an experiment analogous to those performed by Dr. C. J. B. Williams. He tied a glass tube drawn fine at one end into the trachea of a beheaded animal, and when the small end was turned to the flame of a candle he galvanized the pneumogastric trunk; every time he did so the flame was blown, and once it was blown out. The experiment succeeded even when the chest was opened; but the lungs having then collapsed, the efflations were slighter. The movements following the irritations were pulse-like.

*Air-changes in respiration.* Dr. Marchand,‡ from experiments on frogs, confirms the observation of Valentin and Brunner,§ that there is always more oxygen absorbed in respiration than is employed in the formation of the carbonic acid expired. The surplus combines with hydrogen, and forms water. When the animals were deprived of food, less carbonic and more water were

* There is a paper on the anatomy of the lungs by Dr. Eichholtz in Muller's Archiv, H. v, 1845, in which he tries to prove that the nuclei of certain cells, like hepatic cells, which he finds in the lungs, are transformed into blood-corpuscles.

† Wagner's Handworterb. der Physiologie. Art. Nervenphysiologie, p. 586.

‡ Report of the Scientific Meeting at Bremen, in the Medic. Central-Zeitung, Oct. 9, 1845.

§ See last Report.

formed. He found that frogs would not live more than three hours in hydrogen, however pure ; they soon went to sleep, and gradually died.

Dr. Vierordt* has performed on himself a series of 578 experiments to determine, chiefly, the variations produced by different internal and external conditions in the results of the respiratory process. The full evidence for his deductions is given in numerous tables.† He shows, in a first section, the influence of variations in these following conditions: 1. *The time of day.* From 9 to 10 a.m. the pulse becomes less frequent; from 10 to 12 it is stationary; from 12 to 2 it increases fast and much; from 2 to 7 p.m. it regularly falls to the frequency of 9 a.m. In the same periods there are nearly corresponding changes in the number of respirations; but at 7 p.m. they are less numerous than at 9 a.m. There is a slight increase in the quantities of air and of carbonic acid expired in a minute between 9 and 10 a.m.; a slight decrease between 10 and 11: a greater decrease between 11 and 12; a very great increase between 12 and 2 p.m.; and a nearly regular and great decrease from 2 to 7 p.m., at which latter time they are much less than at 9 a.m. The increase from 12 to 2 is probably due to the influence of feeding and digestion, for the chief meal was between 12 and 1, and if it were not taken the respirations diminished before 2 p.m. The other changes also are probably due to variations in other internal processes rather than to those of external circumstances. 2. *The effects of dinner* are to produce an increase, *per minute,* in the pulse of 16·3 beats; in the respirations of 1·72 times; in the air expired of 269 cubic inches; in the carbonic acid expired of 19·37 inches. The evening meal produces similar but slighter effects in all but the frequency of the pulse, which is unaffected by it. 3. *Spirituous drinks,* as Dr. Prout showed, diminish the exhalation of $CO_2$ by $\frac{1}{2}$ per cent., or $\frac{1}{8}$ the whole quantity previously exhaled in a given time; and they do this especially when taken into an empty stomach. 4. *Bodily exercise,* in moderation, makes the exhalation of carbonic acid about $\frac{1}{3}$ more than it is during rest; and for about an hour after exercise there is an increase of about 118 cubic inches in the air expired per minute; and an increase of 14 per cent. in the proportion of $CO_2$ contained therein. 5. *Sleep.* Vierordt has not examined the products of respiration during sleep, but he fully confirms Dr. Prout's observation that there is a great, though quickly transitory, increase in the exhalation of $CO_2$ directly after, or even in the act of, waking. 6. *A change of external temperature* equal to 10° F. produces the following changes in the rates per minute: in the number of respirations, ·28; in the quantity of air expired, 33·4 cubic inches; in the carbonic acid expired, 2·114 cubic inches, or ·0183 per cent. These changes are by increase when the external temperature diminishes, and by diminution when it increases. The temperature in which the observations were made ranged between 38·7 F. and 75·7 F. Within this range the pulse is unaffected by the changes. 7. *A rise in the barometer* equal to half an inch increases the rate per minute, of the pulse, 1·3; of the expirations, ·74; of the quantity of air expired, 230 cubic inches; and, as Dr. Prout showed, diminishes the relative proportion of $CO_2$ expired by ·3 per cent. The barometric pressures in which the observations were made ranged between 29·3 and 30·2 inches. The influence of changes of atmospheric pressure was greater when the temperature at the time was high.

In a second section Dr. Vierordt discusses the influence of *variations in the respiratory movements* on the products of respiration: 1st, *In various frequencies of respiration* the proportionate quantities of $CO_2$ in the expired air are, with six respirations in the minute, 5·528 per cent; with 12, 4·262; with

* Physiologie des Athmens; Karlsruhe, 1845, 8vo.

† The mode of investigation is also detailed; but, as it was the same in all the experiments, it is unimportant to notice it here. Moreover, I have noticed only the facts obtained by his investigations; but the work contains, besides these, many useful deductions from the labours of others.

24, 3·355; with 48, 2·984; with 96 respirations in the minute, 2·662 per cent.* But the absolute quantities of $CO_2$ exhaled in a minute in the same circumstances are (since by rapid breathing the whole quantity of air that is breathed is proportionally increased) as follows: with 6 respirations per minute, 171 cubic inches; with 12, 246 cubic inches; with 24, 396 cubic inches; with 48, 696 cubic inches; with 96, 1296 cubic inches. 2d. *By increasing the volume or depth of the respiration,* the per centage proportion of $CO_2$ in the expired air is diminished; in the deepest respiration it is 1·97 per cent. less than in ordinary breathing. But for this proportionate diminution also there is a full compensation in the greater total volume of air which is thus breathed; for equal volumes of expired air, whether breathed slowly or quickly, deeply or not, contain equal quantities of $CO_2$. 3d. It appears, moreover, that if the air in one ordinary expiration contains on an average 4·48 per cent. of carbonic acid, the air expired in the first half of the whole expiration contains ·76 per cent. less than this average, and that expired in the last half contains ·96 per cent. more than the average, proving that the air in the parts of the lungs nearest the great bronchial tubes contains less $CO_2$ than that in the deeper and more distant parts does.† 4th. *In holding the breath,* the proportion of $CO_2$ in the air within the lungs increases greatly, but in a diminishing ratio; in one minute's holding it becomes 2·42 per cent., and in 100 seconds 3·08 per cent. more than it is in an ordinary expiration. In this condition also the composition of all the air within the lungs very soon becomes uniform.

Finally, Vierordt shows how wide the range of occasional variation is within which, even in health and perfect bodily rest, the several parts of the respiratory functions are discharged. In these conditions the pulse ranged, in occasional instances, between 54 and 101 per minute; the respirations between 9 and 15; the expired air between 1637 and 3676 cubic inches; the carbonic acid therein contained, between 3·358 and 6·22 per cent.; the volume of each expiration between 144·6 and 275 cubic inches.

The changes in the quantity of carbonic acid exhaled in much higher and lower temperatures than Vierordt observed them in have been investigated by M. Letellier.‡ The experiments were performed on small birds, mice, and guinea pigs, who were made to respire for periods varying from half an hour to six hours. The results are stated in tables which show generally that the quantity of carbonic acid exhaled at a temperature between 82° and 104° was about as much below the average at ordinary temperatures as the quantity exhaled at temperatures about zero were above the same average; and the greatest quantity exhaled at the lower temperatures was about twice as much as the smallest exhaled at the higher temperatures. None of the animals experimented on could live without danger in a temperature equal to that which is natural to their own bodies; and all speedily died when the temperature was increased to 5° higher. At temperatures from 80° to 92° they lived and breathed quite tranquilly.

Hannover,§ also, has instituted experiments to determine the quantities of carbonic acid exhaled in certain diseases, and finds, 1, that chlorotic females exhale an absolutely larger quantity of it than healthy ones do; 2, that the quantity is much diminished in all cases of phthisis; and, 3, that it is little or not at all changed in bronchitis.

*Asphyxia.* Many interesting matters connected with the physiology of respiration are well discussed and illustrated by Mr. Erichsen,|| in his essay on

* The author hence deduces a general rule for calculating the proportions, in which, however, he is probably premature.

† See, on the anatomical adaptation of the lungs to this condition, Mr. Rainey's Observations, at p. 553.

‡ Annales de Chimie et de Physique, Avril 1845, p. 478.

§ De Quantitate . . . acidi carbonici ab homine sano et ægroto exhalati; Havniæ, 1845, 8vo.

|| An Experimental Inquiry into the Pathology and Treatment of Asphyxia; Edinburgh, 1845, 8vo.

Asphyxia. 1. To show that, although the cessation of the respiratory movements is not the sole cause of the cessation of the circulation in asphyxia, yet their continuance enables it to be continued longer than it will after they have ceased, artificial respiratory movements with a small quantity of air (a quantity too small to retard materially the occurrence of asphyxia,) were maintained in a dog till all the chemical respiratory changes had ceased, and the heart was motionless. On examination after death, the difference between the quantities of blood in the two sides of the heart was not nearly so great as in ordinary cases of asphyxia: the left cavities contained nearly as much blood as the right; proving that the respiratory movements, unassisted by the chemical changes, had for a time facilitated the passage of blood through the lungs. 2. To show that the arrest of the blood in the small vessels in asphyxia is not the consequence of the action of venous blood in the nervous centres, three dogs were so arranged that while one of them was being asphyxiated by ligature of the trachea, arterial blood might be propelled through its carotids from the carotids of the other two. Care was also taken to prevent the passage of venous blood through the vertebral arteries of the asphyxiated dog, and to prevent congestion of its nervous centres by opening one of its jugular veins. The result was that the dog was asphyxiated in the ordinary time, and that there was the usual amount of congestion of its vessels, although arterial blood had, throughout the experiment, circulated through its nervous centres.

3. Two experiments are related in evidence that, if the action of the heart be maintained, black blood may be propelled by it through a lung long after the chemical respiratory changes have ceased. Dogs were pithed, and while artificial respiration was being maintained the right bronchus was tied. As long as the heart's action continued (and it continued much longer than in ordinary cases of asphyxia), there flowed nearly as much black blood through a right pulmonary vein as of red blood through a left one.

4. A description is given of the contraction of the small arteries, and distension of the veins of the mesentery during asphyxia, as observed with the microscope. And this contraction is maintained to be the principal or sole agent in the obstruction to the passage of the blood observed in asphyxia, the black blood being regarded as a stimulant to the contractile coats of the small systemic arteries and pulmonary veins. [But the description does not disagree with the more probable opinion that the small arteries of the mesentery contracted only because less blood was impelled into them.]

Mr. Erichsen's conclusion from these and some other previous experiments* is that the cessation of the circulation in asphyxia depends upon all three of those circumstances, to each of which, by various former writers, it has been exclusively ascribed: viz., 1, the arrest of the respiratory movements; 2, and more importantly, the weakening of the heart's action in consequence of the lessened quantity and altered quality of the blood which passes into the left ventricle, and the coronary arteries; 3, obstruction to the blood in its passage through the small vessels. [*i. e.* as he thinks, by the refusal of the minute pulmonary veins and systemic arteries to receive venous blood, but, as others believe, because the capillaries cannot transmit blood charged with carbonic acid.]

The average periods, in minutes and fractions thereof, at which the chief phenomena of asphyxia are observed in dogs are stated thus: voluntary movements cease in $1\frac{3}{4}$; involuntary in $2\frac{1}{2}$; the blood in the arteries becomes as black as that in the veins in $1\frac{3}{4}$, or, occasionally, in $1\frac{1}{4}$; ventricular contractions cease in $9\frac{1}{2}$ (at the earliest in $6\frac{1}{2}$, the latest, in adult animals, in 14); the left auricle ceases to act in 18 minutes, the right in $19\frac{1}{2}$, but once, in an adult animal, the former continued acting for 37, and the latter for 44 minutes;

* Medical Gazette, 1842.

pulsations in the femoral artery continue for 7½ minutes. In puppies four days old the ventricles contracted regularly for 117 minutes, and with irregular movements for three hours; and the auricles continued acting for three hours and twenty-five minutes.

ANIMAL HEAT.

Liebig* has shown the error of the opinion derived from Dulong and Despretz, that the quantity of heat generated by an animal which consumes by respiration a certain quantity of oxygen in a given time, is less than would be produced by the direct combustion of carbon and hydrogen in the same quantity of oxygen. He proves that this opinion is founded on incorrect premises; the combustion-heats of carbon and hydrogen having been reckoned too low by both Dulong and Despretz; and having established the authority of new and more accurate numbers to represent these combustion-heats, he shows that the amounts of heat developed by animals consuming certain proportions of oxygen, are nearly equal to those which are produced by the direct combustion of definite proportions of carbon and hydrogen in the same quantity of oxygen.

For the sake of their relation to the foregoing observations of Vierordt, I place here some of these by Gierse,† whose work should have been noticed in the Report for 1842-3. At different periods of the day, he found the temperature under his tongue as follows:—from 11 P.M. to 2 A.M. 98·26° F.; from 6 to 8 A.M, before breakfast, 98·55° F.; from 6 to 8 A.M on other days, after breakfast, 98·73°; between 9 and 11 A.M., 99°; just before dinner, 98·82°; after dinner, 99·5; between 3 and 6, P.M., 98·61°; between 6 and 10 P.M., 98·86°; from 11 P.M., to 1 A.M., while asleep, 98·15°. All the other observations by this author relate to the temperature of inflamed parts, except a few from which he believes that there is no increased heat in the vagina during menstruation or parturition. He found almost constantly that an inflamed part communicated its heat to the thermometer more rapidly than a healthy part of the same temperature did. He found also that, in animals, struggling and excitement commonly raised the temperature more than the inflammation of a part did.

Some of these results also, are confirmed by Dr. John Davy.‡ He finds the temperature highest in the morning, on rising after sleep; high, but fluctuating, till the evening; and lowest about midnight, ranging from 98·7° to 97·9°. In active exercise, within the limits of exhaustion, the temperature of the body is increased in direct proportion to the exertion; in passive exercise in the cool air it is lowered; during quietude in an atmosphere from 42° to 32°, it may be reduced from 1° to 2°. Sustained mental exertion slightly raises the temperature; a light meal scarcely alters it; a heavy one lowers it.

Sundry observations are recorded by Dr. Dowler and other American surgeons§ of considerable increase of temperature of the surface of the body after death from yellow fever and coup de soleil. Within a few minutes after death the temperature is said to rise to 102°, and then to increase steadily to 106° or 108° or even to 113°. Thus it may remain for from four to six hours, being usually higher at the thigh and abdomen than in the heart; then it gradually subsides to the temperature of the surrounding medium. The cases are said to have all occurred in the summer, in a temperature of at least 80°; but they are very imperfectly related. In the paper first referred to in the subjacent notes, another phenomenon is mentioned, which is certainly very rarely if ever observed in this country, namely, the maintenance of the power of forcible contraction in the voluntary muscles for six or seven hours after death. When all the muscles of the fore-arm are dissected bare (the arm

* Lancet, Feb. 22, 1845, and Annalen der Chemie und Pharmacie, Jan. 1845.

† Quænam sit ratio caloris organici, Dissert. inaug.; Halæ, 1842, 4to.

‡ Proceedings of the Royal Society, No. 61, June 19, 1845.

§ B. Dowler (New Orleans), in the Boston Medical Journal, Aug. 6, 1845; in the New York J. of Medicine, Sept. 1845; in the Medical Examiner, Sept. 1845.

being amputated at the shoulder) a blow with the hand will excite powerful contractions in the bared muscles; and twenty minutes or more after death a large hatchet weighing three pounds being tied in a subject's hand, it is said that the fore-arm was bent several times so as to raise the hatchet and strike the trunk as often as the flexor muscles were struck with the hand.

### DIGESTION.

STRUCTURE OF THE DIGESTIVE ORGANS; *The Tongue.* Dr. Nuhn* has minutely described a pair of glands in the substance of the lower part of the apex of the tongue. Each gland lies about two lines from the median line, just below the ranine artery, on the outer side of the expanding branches of the lingual division of the trifacial nerve, under some longitudinal fibres of the anterior part of the stylo-glossus. It has the structure of a salivary gland, and measures from 7 to 10 lines in length, from 3 to 4½ in breadth, and from 1½ to 2½ in thickness. It has, at least, five ducts, which open through the mucous membrane over it by small orifices, each surrounded by a slightly elevated ring; it receives many branches from, and sometimes surrounds, the ranine artery. Dr. Nuhn has at present found the gland in man and the orang alone; he has sought it in vain in many other mammalia; he thinks therefore it may be a mucous gland whose fluid facilitates the movement of the tongue in speaking: [it looks exactly like a salivary gland].

*Structure of the Palate.*† Dr. Tourtual‡ has detected a new muscle of the posterior nares and palate, and found it regularly in six men as well as in dogs, cats, and sheep. It lies between the mucous membrane and the internal pterygoid plate, before the Eustachian tube, and behind the inferior meatus of the nose. Its upper edge or origin extends from below the palatine process of the turbinated bone backwards and upwards, towards the upper margin of the Eustachian tube; in this line the muscle arises from the vertical plate of the palate bone, and from the internal pterygoid plate of the sphenoid, reaching sometimes to the cartilage of the Eustachian tube; hence its fibres straight descend, and are augmented by others arising from the several parts near which they pass; and going just behind the hard palate, they enter the anterior and outer part of the soft palate, in which they expand on an aponeurosis, mingling with that of the circumflexus palati. The posterior margin of the muscle is usually confused with the anterior fibres of the levator palati, and is covered by a short fold of mucous membrane, which forms the anterior margin of the orifice of the Eustachian tube.

The discoverer proposes for this muscle the name of pterygo-palatine, or levator palati mollis anterior seu minor.§ It receives a (probably sensitive) filament from the internal palatine branch of the fifth; and has, probably, also motor fibres from the glosso-pharyngeal. The function of the pair of muscles is probably that of elevating and of slightly stretching transversely the *anterior* and lateral parts of the soft palate, for which the larger and posterior levatores palati do not provide. In this tension of the palate they probably prevent the anterior fibres of the circumflexi from drawing the anterior part of the palate backwards. They probably are important in sounding the palatine consonants, and their posterior portions may assist the greater levatores palati in narrowing the Eustachian orifices.

*Structure of the Liver.* An elaborate anatomy of the liver, with measure-

* Ueber eine bis jetzt noch nicht näher beschr. Drüse im innern der Zungenspitze; Mannheim, 1845, 4to. I supposed he had discovered these glands, but Schlemm (Müller's Archiv, H. v, 1845) shows that they were discovered by Blandin, and decribed in his Anatomie Topographique, p. 175; Paris, 1834.

† On the Structure of the Teeth; see, in a future part, Prof. Owen's account of their development.

‡ Müller's Archiv, 1844, Heft v, p. 452.

§ This name had better be used; for the former has been already applied to what is probably a part of this muscle, arising from the hamular process of the pterygoid plate, and mingled with fibres of the pterygo-pharyngeus. See Haller, Elem. Physiol. t. vi, p. 80, note *t*.

ments of all its measurable parts, is published by Theile.* He confirms Mr. Kiernan's account of its lobular structure; for though he admits that the investment of each lobule, which is so dense in the pig and some other animals, may be absent or very thin in the human liver, yet he urges, and very rightly, that the latter is, by the arrangement of its vessels, divided into lobules, just like those of the pig and others. For in the human liver, as in theirs, each portion thus bounded by vessels, if not by a distinct capsule, is a miniature liver, having in itself a complete bile-secreting apparatus. He confirms also the account of the hepatic cells being arranged in networks with a radiated plan; but he denies the existence of the vaginal branches and plexuses of the portal vein as described by Mr. Kiernan. These vaginal plexuses, he says, "are derived, not from the portal veins, but from the hepatic arteries, from which they are completely filled when both arteries and veins are at the same time injected; and when they appear to be injected from the portal vein, it is because the injection has passed through those vessels by which the blood of the hepatic artery is carried to the portal vein. The interlobular portal veins are therefore, he says, derived directly from the portal veins; and those which appear to be vaginal branches of the portal vein are its *internal roots*, by which it receives the blood which has served for the nutrition of the hepatic ducts and other vessels of the liver. But that which is most new in his essay is the account of the glands of the biliary ducts. The orifices of these are, as Mr. Kiernan showed, arranged in two opposite rows through the whole extent of the ducts within the liver, as far as they can be dissected; an arrangement peculiar to the human liver. Their number is less in the human than in the other livers which Theile examined, but their form is more complex. They generally consist of an elongated tortuous canal, bearing alternate small sacculi and pedicled bunches of cellules; reminding one of the Meibomian glands. These canals, moreover, branch, and their branches anastomose with one another, and with those of the adjacent glands. Such a net-like connexion of the gland-canals occurs within the Glisson's capsule, investing the larger and middle-sized hepatic ducts; but it is most remarkably developed in the transverse fissure of the liver. Here, after the hepatic duct has been well injected, red streaks, forming plexuses, can be seen, which are nothing but large examples of these glands.† The canal-shaped glands in this situation are a quarter of a line in diameter, (in other parts they are from one fifth to one tenth); their walls are beset by pouches and bundles of sacculi, some of which are one sixth of a line in diameter, and there open into them at short intervals elongated glandules, just like those found in the prolongations of Glisson's capsule, except in that they do not open directly into the hepatic ducts.

Krause‡ suspects that these glands of the hepatic ducts, as well as the vasa aberrantia of Weber, are incompletely injected bile-ducts, [or, perhaps, they should rather be considered as such ducts imperfectly developed; for if Krause's account of the acinous structure of the liver be true, no line of essential distinction could well be drawn between them and the more perfect ducts]. He holds still§ that all the bile-ducts have acini at or near their ends, having confirmed his opinion by recent numerous injections. He agrees generally with Kiernan and Weber's account of the reticular arrangement of the minutest hepatic ducts, having often seen this by injection; and he adds that, on many of those ducts he has injected, and has seen, after dissolving the injection that was in them, regular round and oblong vesicles or acini, from $\frac{1}{730}$ to $\frac{1}{480}$ of an inch in diameter; many of these acini, grouped upon their several ducts, compose each lobule, each group of acini having one duct, and therefore each lobule having probably several ducts, or roots of the hepatic

* Wagner's Handwörterbuch der Physiologie, art. Leber.

† E. H. Weber has described these as a part of the vasa aberrantia of the hepatic ducts, similar to those in the left lateral ligament and some other situations. See last Report, p. 29.

‡ Müller's Archiv, Heft v, 1846.

§ His former paper is in Müller's Archiv, 1837; and the same account is given in his Handbuch der Anatomie.

ducts, which proceeding from it enter into the lobular plexus of ducts. He says also, that the acini may often be discerned entire at the edges of very thin sections of the human liver; they appear within the lobules as regular, round, or oval, yellowish-gray and very thin-walled corpuscles or cells, invested by very fine and short fibro-cellular fibrils, and each containing from six to eight hepatic cells, with, in many cases, bile.

M. Natalis-Guillot* has also, (if I rightly apprehend his expressions), confirmed by injections the account given in the last Report of the retiform arrangement of the minutest bile-ducts. These, he says, surround, either in a net, or in dense tufts, the whole surface of each lobule, and spread over the surface of each of the "ultimate ramifications of the vena portæ," i. e. of the interlobular portal veins. The ducts themselves, he thinks, are surrounded by minute branches of the hepatic artery.

Properties of the digestive fluids. *Saliva.* Experiments by M. Magendie† and others on the saliva of the horse have shown a difference between that secreted by the parotid gland and the mixed saliva from all the glands. The parotid saliva obtained from a fistula into the duct is more alkaline than the rest [as Mitscherlich also showed]. It contains carbonate [?] of potass which the rest does not contain, and a much larger proportion of ptyaline and albumen, the latter constituting one fifth of the whole solid mass. The parotid saliva also has no influence on starch-paste or raw starch, at a temperature from 104° to 167°; but the mixed saliva speedily transforms the starch-paste into sugar at 104°, and at the same temperature acts slowly but evidently on raw starch and coagulated albumen. This mixed saliva, obtained from food, masticated and swallowed into the œsophagus, is not limpid and clear like that from the parotid, but yellowish-gray, easily becoming turbid. It is weakly alkaline, and contains no carbonate, but abundant alkaline chlorides. There is a great "analogy" between the different kinds of saliva poured into the mouth of man, and those just described as secreted in the horse.

*Gastric Fluid.* The researches of M. Blondlot,‡ from which he deduced the presence of an acid phosphate of lime in the gastric fluid, are partly confirmed and partly opposed by those of Dr. R. D. Thomson.§ Thus, like Blondlot, he has never found a volatile acid in the stomach if digesting animal food alone; and he disproves the presence of hydrochloric acid in the gastric fluid during the digestion of either kind of food. But, contrary to Blondlot, as well as to the experiments next mentioned, he says, that the acid of the stomach can be saturated with chalk; and that therefore it cannot be an acid phosphate of lime. What the acid is, his experiments leave uncertain; but in the digestion of vegetable substances, he finds a volatile acid always present in very minute quantity, and a fixed acid which, he says, resembles the lactic more nearly than any other.

MM. Bernard and Barreswil hold that the gastric acid is the lactic. Their experiments,‖ like those of M. Blondlot, show that it cannot be either the acetic or hydrochloric—the first cannot be distilled from it; and, though the second can, yet it is only at the last, when the chloride salts are decomposed by the acid really present. Moreover, a minimum of oxalic acid produces a precipitate of oxalate of lime, which it could not do if even $\frac{1}{2000}$ of free hydrochloric acid were present. But they show that M. Blondlot failed to observe effervescence on adding carbonate of lime to the gastric fluid, because of the state of dilution of the fluid: when it is concentrated by evaporation distinct effervescence ensues. This effervescence proves the existence of some acid besides the phosphoric; yet, since the addition of an excess of carbonate of lime cannot completely neutralize the acid, it is highly probable that a small

* Comptes Rendus, 18 Novembre, 1844.

† Archives Gén. de la Médecine, Nov. 1845, from the Acad. des Sciences, 20 Oct.

‡ See last Report, p. 24.

§ On the digestion of vegetable albumen, &c. Philos. Mag., May, 1845; and Lancet, May 17, 1845.

‖ Comptes Rendus, 9 Decembre, 1844.

proportion of phosphoric acid free, or in an acid salt, is present in the gastric fluid and gives it part of its acid reaction. The other acid with which the carbonate of lime effervesces is, the authors believe, the lactic; and they show that in every essential respect the conduct of the gastric fluid with various tests is similar to that of water acidulated with lactic acid, and with the addition of a little chloride of sodium.

The experiments also of M. Melsens* confirm some of these, proving that both marble and other carbonates of lime lose weight when immersed in gastric fluid, and that there must therefore be a free acid in it.

*Bile.* The most careful examinations of the urine and blood of a patient with intense jaundice did not enable Scherer† to detect in either of them, a trace of any constituent of bile except the colouring matter and cholestearine. In evidence of the speedy transformation which the biline would probably undergo in the blood, he mentions that in a large quantity of green fluid vomited, and containing abundant biliary colouring matter, he could not detect a trace of the biline which it must previously have contained. In the same essay he gives an accurate account of his analysis of the biliary colouring matter which he collected from the patient's urine.

The conclusion respecting the non-existence of the essential principles of the bile in the fæces is confirmed by the delicate test for bile invented by Pettenkofer.‡ To the fluid supposed to contain bile ⅔ of its volume of sulphuric acid are added by drops, that the temperature may not rise above 140° F., and then from two to five drops of a solution of sugar (one to four parts of water). Presently a reddish violet colour appears, intense in direct proportion to the quantity of bilic acid. By this test no bile (except the colouring matter) could be found in healthy fæces; but the fæces of diarrhœa and those discharged after purgatives contain complete bile. So also, by this test, bile could always be found in the urine of the pneumonic.

Dr. Redtenbacher§ has found that taurine contains 26 per cent. of sulphur, and his discovery is confirmed by Dr. Gregory.||

*Pancreatic fluid.* Twenty-eight grains of the pancreatic fluid of an elephant, collected eight days after death, yielded to Professor Bergmann,¶ of Bonn, 92·77 per cent of water and 7·33 per cent. of albumen, caseine, and a minute quantity of chloride of sodium and carbonate of soda.

Digestive Principles. MM. Barreswil and Bernard** also maintain that the active organic digestive principle (presently again to be mentioned) is the same in the saliva, the gastric fluid, and the pancreatic secretion; and that the special action which it appears to exhibit in these several fluids is due only to its being combined with an acid in the gastric fluid, and with an alkali in the others. They say that from whichever of these three sources this principle is obtained it will, if an acid be added to it, digest meat, gluten, and other azotised compounds, and act like artificial gastric fluid; but if it be made alkaline it will only be capable of digesting the amylaceous principles, and thus will be an artificial saliva or pancreatic fluid. So also, by making gastric fluid alkaline, it will act like saliva or pancreatic fluid; and by making either of the latter acid it will act like gastric fluid.

Process of Digestion. The principal researches of the year to be placed under this head have had reference to the digestion of the saccharine and amylaceous principles of food. The most considerable are those of MM. Bouchardat and Sandras,†† whose results are as follows:

* Comptes Rendus, 9 Decembre, p. 1289.

† Annalen der Chemie und Pharmacie, Marz, 1845; and Lancet, May 24, 1845.

‡ Annalen der Chemie u. Pharmacie, No. lii, p. 90.

§ Annuaire de Chimie, p. 724, from the Comptes Rendus, t. xx, 1845.

|| Outlines of Chemistry, Part ii, p. 566.

¶ Oesterr. Med. Wochenschr., Dec. 14, 1844, from the Med. Corresp. Blatt Rhein. u. Westphal. Aerzte, No. 17, 1844. Much more concerning the nature of the pancreatic fluid will be found in the following paragraphs.

** Gaz. Méd., 12 Jul. 1845, from the Report of the Acad. des Sciences, 7 Jul.; Comptes Rendus, 25 Jul.

†† Report from the Académie des Sciences, 20 Jan. 1845, in the Gazette Médicale, 25 Jan. 1845.

When cane-sugar is given to dogs it is found, either unchanged or converted into *sucre interverti* or lactic acid, in the whole length of the digestive canal. After it has been given for several days it may be found in the urine, and in the bile, blood, and chyle. When cane-sugar is introduced directly into the blood it passes into the urine; but when the same quantity of *sucre interverti* or lactic acid, was so introduced none was detected in the urine. In order that cane-sugar may be destroyed in the blood (i. e. ultimately reduced to carbonic acid and water), it must first be transformed into *sucre interverti*, or lactic acid in the digestive canal.

Raw starch is very imperfectly digested by men and carnivora. The greater part of it may be found unaltered in the excrements. A greater effect is produced on it by the digestive organs of herbivorous rodents. It undergoes no change in their stomachs; but in the contents of their small intestines (which are always alkaline, except sometimes at the pyloric part of the duodenum) there are found, together with entire starch granules, others that are cracked, eroded, or almost wholly destroyed; and, also, dextrine with traces of grape-sugar. The cæcum contains an acid paste, in which there are some entire starch granules, dextrine, grape-sugar, and lactic acid. The same materials are found in the rectum. The dextrine, grape sugar, and lactic acid may also be detected in the blood and the bile, but not in the urine of these animals; and the blood of their vena portæ contained more water and more of these three substances than their arterial blood did—making it sure that they are absorbed by the blood-vessels, not by the lacteals.

Graminivorous birds digest raw starch more completely than mammalia do. It undergoes no change in their crops; and in their gizzards, though there are traces of dextrine and grape-sugar, yet nearly all the starch-granules appear unchanged. In the small intestines the starch-granules are gradually more and more destroyed, and ultimately they undergo the same changes as in the intestines of the herbivorous rodents, but more completely. In both, the authors consider the changes to be due to the high temperature, the alkaline reaction, and the presence of a secreted principle which acts like diastase, only with less energy: all which conditions exist in greater force in the birds than in the mammalia.

When the starch-granules have been burst by cooking they are digestible by men and carnivorous animals. Their solution commences in the stomach, and is slowly continued through the intestinal canal. The products are dextrine, grape-sugar, and lactic acid; which are found mixed with some starch remaining unchanged.

If more than a certain proportion (one gramme, at the most, for an adult dog) of a feculent or saccharine principle be mingled at once with the blood, sugar is always eliminated by the kidneys. Hence the slow introduction into the blood of the products derived from the digestion of these principles appears to be an essential condition for their due disposal; and this condition is secured by two chief means, namely, the slowness of their solution, and the chief manner of their absorption. They are formed into soluble compounds in the intestines; and these being absorbed by the blood-vessels, are carried to the liver, where, if combustible matters are in excess in the blood, the greater part of them are secreted and discharged with the bile into the intestines, whence some of them may be absorbed with the other soluble constituents of the bile. "Thus then is established a limited circulation of the combustible matter, which, by this admirable artifice, is only gradually carried into the general circulation."

These conclusions concerning the transformations which starch undergoes in digestion are, to some extent, confirmed by Dr. R. D. Thomson,* whose experiments show that dextrine and soluble starch exist in the stomachs of pigs fed on farinaceous food during and for some time after digestion, and

* Philos. Mag., May 1845; and Lancet, May 17, 1845.

that sugar exists in the blood of the same animals in the proportion of from 2·57 to 8·05 grains in 1000 grains of the serum.

In a subsequent memoir* MM. Bouchardat and Sandras state that the principle which, as above-mentioned, appears to act like diastase in the transformations of starch is secreted chiefly by the pancreas. They find the pancreatic fluid of birds transparent, viscid, slightly alkaline, and capable of liquefying starch-paste, and of transforming it into dextrine and grape-sugar. Portions of pancreas cleared of blood and large vessels possess the same power in a very high degree; and no other organ besides the pancreas, and in a slighter degree the salivary glands, possesses such a power.† It is, moreover, wholly destroyed both in the pancreatic fluid and in the pancreas itself by such influences as destroy the like property in diastase, such as a temperature of 212°, tannin, mineral acids, metallic salts, &c.

The same influence which these authors ascribe to the pancreatic secretion is ascribed by M. Mialhe‡ to the saliva, from which he gives directions for obtaining the digestive principle, *animal* or *salivary diastase*,--by filtering it and then treating it with five or six times its weight of absolute alcohol. The diastase being insoluble in alcohol is thus precipitated in white flocculi. He describes the aqueous solution of this substance as insipid and neutral, not precipitable by subacetate of lead, and when left to itself undergoing a transformation into butyric or some similar acid. With raw starch this salivary diastase requires several days for the production of dextrine and sugar of starch; but with starch-powder the change is quickly effected; and with starch-paste it is very speedily completed if aided by a temperature of about 160°.§

The experiments of M. Lassaigne|| confirm those of MM. Bouchardat and Sandras as to the properties of the pancreas and its fluid; and, at least in great measure,¶ those of M. Mialhe on the properties of the saliva. He shows that at the natural temperature of the body saliva has no effect on whole starch, and that mastication does not change the form in which it naturally exists in cereal grains; that horse's saliva does not act on starch even when its grains are broken; but that human saliva, though it does not affect raw and whole starch at a temperature of 100°, can even at a temperature of from 64° to 68° convert powdered starch partly into dextrine and partly into sugar of starch; the envelopes of the granules preserving at the same time their property of becoming violet when touched with iodine.

*Influence of the Bile in digestion.* Dr. Platner** has made experiments to find how the bile contributes to digestion. He has confirmed, what Simon and others showed, that the fæces contain none of the bile except its colouring matter [and some of its fat?]; and what Purkinje showed, that bile will put a stop to or prevent the artificial digestion of coagulated albumen. On mixing pure artificial digestive fluid, neutralized by carbonate of soda, with bile, no change took place; but on adding hydrochloric acid to the mixture it became very turbid. The same happened when bile was mixed with digestive fluid not neutralized; but hydrochloric acid added to bile alone produced no precipitate. The precipitate consisted of bilic acid united with some organic body, perhaps pepsin, explaining probably the fact quoted above from Purkinje.

When bile was added to a solution of albumen in acetic acid, a precipitate

* Archives Gén. de Médecine, Mai 1845; Report from the Acad. des Scienees, 14 Avril.

† See in connexion with this subject a paper by M. Bouchardat, " Sur la fermentation saccharine ou glucosique," in the Annales de Chimie et de Physique, Mai 1845, t. 89, p. 61.

‡ Ibid., 5 Avril 1845; Report for the 31st of March.

§ According to Dr. R. D. Thompson (l. c.) the transformation of starch into dextrine is effected to some extent by boiling it for half an hour in pure distilled water.

|| Arch. Gén. de Médecine, Mai 1845, from the Report of the Acad. des Sciences, 7 Avril.

¶ Ibid. Juillet 1845, from the Report of the Acad. des Sciences, 2 Juin. In the earlier paper his results were opposed to those of M. Mialhe.

** Muller's Archiv, 1845, Heft iv. He has since published a special work, " Ueber die Natur und den Nutzen der Galle," Heidelberg, 1845; but I have not yet received it. Another work relating to the physiology of the bile is H. Meckel, De genesi Adipis; Halis. 1845, 8vo.

was formed which was insoluble in all acids, but soluble in alkalies. When bile and albumen were mixed and acetic acid added, a precipitate like coagulated fibrine was formed; and a similar precipitate was formed by the agency of even carbonic acid; showing that although the bilate of soda (i. e. the pure principle of bile) retains its composition under the action of either acids or alkalies alone, yet it is decomposed easily by combinations of acids with organic substances. When bile was added to a solution of albumen or gelatine obtained by artificial digestion, precipitates were formed which were soluble in acetic acid and consisted of bilic acid united with the organic substance. Sugar and gum in like circumstances appeared to unite with the fatty matters of the bile.

*Fæces.* The usual microscopic constituents of human fæces are thus enumerated by Dr. Gobee.* 1. A large quantity of vegetable cellular tissue, with or without epidermis and hairs. 2. Vegetable hairs. 3. Vegetable spiral vessels. 4. Elongated quadrangular plates of light yellow colour in great abundance, of uncertain nature; they are not affected by acetic acid, and are insoluble in cold ether, but iodine displays transverse striæ on them. [Probably they are portions of muscular fibre. I have found such, tinged pale yellow by the bile, in the fluid discharged through an artificial anus.] 5. Large quantities of crystals of phosphate of ammonia and magnesia. 6. Fat-globules or cells in various quantity. 7. A great quantity of granules. 8. Few epithelium- and mucus-cells. 9. Much of the brown-colouring matter of the bile.

### ABSORPTION.

*Structure of the Lymphatics.* The subject of one of Mr. Goodsir's excellent essays† is the structure of the lymphatic glands. At the points of connexion between the extra- and intra-glandular lymphatic vessels, the coats of the former (whether afferent or efferent) separate. The outer coat is continued into the external capsule of the gland, from which processes pass inwards, binding together the substance of the gland, and supporting the vessels within it. The middle, or fibrous coat, is usually nearly lost, as the vessels pass towards the centre of the gland. But the internal coat becomes thicker and more opaque in the intra-glandular lymphatics; and when, in any of these thickened, dilated, and oft anastomosing vessels, it is broken up, it appears composed of two substances; namely, first, a thin transparent membrane, in which ovoidal bodies, containing one or more minute vesicles, are imbedded, as "germinal spots,"‡ at regular distances; and secondly, thick layers of close-packed spherical nucleated particles about 1-5000th of an inch in diameter, which make the vessel appear opaque, and leave only a narrow and irregular canal along its axis, the walls of which canal appear formed by them, not by any membrane lining them. The capillaries in the lymphatic glands ramify in contact with (not in the substance of) the external layer of their coats; as they do on the ultimate ducts of the true secreting glands; and they form as fine a network.

The general result of these observations is plainly favorable to the opinion of an intimate analogy between the lymphatic and the true secreting glands; of which some account was given in the last Report, and more will presently be said in speaking of the glands without ducts.

*Process of Absorption.* Some observations by E. H. Weber§ are said to prove that the chyle is first absorbed into the epithelium-cells covering the villi, which, at a certain period, are found full of chyle-globules, and from which it is transferred into the proper cells of the villi, to be by them conveyed to the lacteal vessels. And among the cells of the villi, it is said that

* Kliniek; Tijdschrift voor wetensch. Geneeskunde; voor G. C. Gobée, 1844, St. iv.

† Anat. and Pathol. Observations, No. viii, p. 44.

‡ The expression has reference to the author's general theory of nutrition: the bodies he describes appear to me identical in aspect with the nuclei or cytoblasts of many secreting gland-ducts.

§ Archives d'Anat. Gén. et de Physiol., Jan. 1846.

two peculiarly large ones are often seen in man during digestion, which touch each other, and of which one contains an opaque-white liquid, and the other a clear fatty matter.

Experiments on absorption have been performed by Mr. Fenwick,* and their results are very like those obtained by Herbst. 1. He relates two experiments to show that indigo will pass into the lacteals. 2. He relates many to prove that the lacteals do not absorb strychnia, or milk, or other food, from any part of the digestive canal in which the blood is not circulating. 3. He shows, as Herbst does, the passage of liquor sanguinis and blood into the lacteals when the adjacent blood-vessels are much congested. 4. In other experiments, oily matters (?) and prussiate of potash injected into the pleura of a rabbit were shortly after found in the lacteals. 5. Others again show that the action of the lacteals and lymphatics is independent of nervous influence. 6. And others confirm the fact already known, that they continue to propel their contents after apparent death. 7. From his experiments, from analogy, and from many ingenious, but I think insufficient, arguments, Mr. F. concludes that these vessels obtain their fluid neither by absorption, nor by secretion from the blood-vessels adjacent to them, but by parts of the contents of the blood capillaries, according to the degree of congestion, being directly and mechanically effused into them.

*Propulsion of Lymph.* An attempt has been made by Dr. Bidder† to determine the average quantity of lymph and chyle which flow through the thoracic duct in a given time. The measurements were made by collecting what flowed from the thoracic duct immediately after death. In five cats, the fluid continued to flow from one to six minutes, and, judging by the quantity collected in this time, the average quantity which would have flowed in an hour, was 373 grains (the extremes being 276 and 480 grains); and the average proportion between the weight of the cat and the weight of the chyle and lymph, which at the same rate, would have flowed in twenty-four hours, was as 5·34 to 1 (the extreme proportions being as 6·8 : 1, and as 5·1 : 1). In two dogs the average rate of efflux (similarly calculated) was 3858 grains in the hour; and the average weight of the dogs was, to that of the chyle and lymph which would have flowed in twenty-four hours, as 6·66 : 1. Now, the average weight of blood in cats is, to the weight of their bodies, as 1:5·7; and of dogs, as 1:4·5; hence the quantity of fluid daily traversing the thoracic duct of a cat, is about equal to the whole quantity of blood in it; and the quantity of the same in a dog is equal to two-thirds of its blood.

*Lymphatic hearts.* Volkmann‡ has proved that the rhythmical movements of the lymphatic hearts of frogs depend on the direct influence of portions of the spinal cord. They cease on the instant of destroying the cord, though those of the blood-heart continue for many hours. But repeated experiments showed that the contractions of the anterior hearts would continue long while they retained a nervous connexion with the cord about the third vertebra; and those of the posterior hearts as long, if their connexion with the cord at the eighth vertebra was uninjured. The movements thus continued in the lymphatic hearts though the whole of the cord, except these portions, were destroyed; and on the instant of destroying either of these portions, though all the rest of the cord were intact, the movements of the corresponding hearts ceased. Removal of the brain had no influence. The movements continued also after the division of the posterior spinal roots (they were therefore not reflex), but they ceased directly on the division of the anterior roots.

* Lancet, Jan. 11, 18, 25, Feb. 1, 1845. † Muller's Archiv, 1845, Heft i.

‡ Ibid. 1844, Heft iv, and Wagner's Handworterbuch, art. Nervenphysiologie, p. 489. Valentin, in the body of his Physiologie (vol. ii, p. 767), denied the truth of these experiments, but in a later appendix confirms them. For other papers on the lymphatics, see Oesterlen, as presently quoted; H. Nasse, an elaborate article on the lymph in Wagner's Handworterbuch; and papers on some unusual arrangements of the lymphatics, by Svitzer and von Patruban, in Muller's Archiv, H. ii, 1845.

### GLANDS WITHOUT DUCTS.

In the last Report I deferred the notice of Dr. Oesterlen's observations* on the vascular glands that I might include with it that of the then unpublished essays of Mr. Simon.† I am thus able to set out more briefly the results of the two most important works ever yet published on these organs, and this with advantage even to Dr. Oesterlen's work, for his facts gain importance and clearness by the corroboration and bright illustration which they receive from the truly admirable researches of Mr. Simon.

*Thymus Gland.* According to Mr. Simon, the earliest condition of the thymus gland is that of a simple tube of transparent homogeneous membrane, with granular and dotted contents. It presents at regular intervals elongated thickenings of its wall, which are probably the attenuated nuclei of a series of primordial cells, by the fusion of which the tube may be first formed. The tube has no connexion with the respiratory mucous membrane. In the next stage the tube (which remains in the axis of each half of the gland as its central cavity,) bulges at certain points of its length, forming diverticula or follicles, which communicate with and have the same structure and contents as itself. These usually assume hemispherical or pedunculated forms; and, in the next stage, themselves branch or form secondary and tertiary bulgings; and this is generally effected without elongation of the isthmus or pedicle by which the primary follicles were connected to the main canal, so that the secondary ones appear sessile. The progress of the development of the gland consists in repetitions of this process—the growth of follicles extends successively to all parts of the main tube; in each new crop of follicles are repeated the same acts of development and branching; and the whole substance of the gland enlarges by interstitial growth.

The gland continues growing through the whole "age of early growth;" and the period at which it attains its greatest size cannot be more nearly determined than the age of completed growth of the whole body can. Mr. Simon's observations on this point agree with those of Haugsted.

This account of the development, affords some notion of the mature structure of the thymus. According to both Simon and Oesterlen, whose observations now begin to coincide, it consists of a collection of polygonal mutually flattened membranous cells, from half a line to nearly two lines in diameter, the terminal vesicles or follicles of the gland. These are ranged in masses round a common axis; each mass forming a sort of cone, whose apex is directed towards the axis. The vesicles or cells are not completely close and separate, they are closed in about three fourths of their periphery; by the remaining part, each is attached to the general trunk of the glandular substance and opens into some diverticulum of its common cavity.

The walls of the vesicles are formed by the same kind of homogeneous membrane as the primitive tube; each having on its exterior a capillary network; groups of them are connected by investing areolar tissue, in which is mingled a small proportion of delicate elastic fibrils.

The vesicles are filled by a fluid and a multitude of corpuscles. The corpuscles, according to both observers, have the structure and relations of nuclei. They are generally circular, yet often deviate widely and variously from this form, and are flat and disc-like. Their average diameter is $\frac{1}{3830}$ of an inch; they are characteristically dotted, having from two to five very small dark spots, either scattered, or collected into a single corpuscle in their centres. In animals past the most active period of the thymus, there may be found cells in which these dotted corpuscles appear as nuclei, and which are, or become, perfect fat-cells.

* In his Beitrage zur Physiologie; Jena, 1843, 8vo, pp. 1-95.

† A Physiological Essay on the Thymus Gland; London, 1845, 4to; and, The Comparative Anatomy of the Thyroid Gland; in the Philosoph. Transactions, 1844, part 2.

The fluid of the thymus-cells contains (in the active period) proteine-compounds and traces of fatty matter; and the gland itself, on ultimate analysis, yields nearly the same proportion of essential elements as blood and flesh do.

The most important facts afforded by the comparative anatomy of the thymus, laboriously investigated by Mr. Simon are, 1st, that it "belongs, without exception, to all animals breathing with lungs, and to no others." 2. That in the hybernating rodent animals, the large persistent thymus and its prolongations into large masses which lie in the posterior mediastinum, are wholly composed of fat, the nuclei or cytoblasts which its cells contained having, at the approach of the hybernating period, genuine fat-cells formed round them. 3. That the thymus of birds, hitherto unknown, is a semitransparent ampullated tube, following the line of the superficial cervical vessels, and very early ceasing to discharge its functions. 4. That in reptilia, to which also a thymus was generally denied, its existence is constant. In the hybernating serpents also there is always appended to the persistent thymus, an accessory organ or fat-body. In the batrachia and some others, the thymus is at a very early period converted into a mass of fat, which is persistent. The larvæ of batrachia while breathing with gills have no thymus; its development begins as soon as their pulmonary respiration is established. Among the icthyoid reptiles with persistent gills, it is found in the menopoma, amphiuma, axolotl, and menobranchus, but not in the siren and proteus. 6. In fishes, a thymus is not found.

*Thyroid Gland.* According to Mr. Simon, the thyroid gland consists of a dense aggregation of completely closed vesicles, each formed by a layer of delicate homogeneous membrane (*limitary membrane*), invested by a close capillary network. These are the analogues of the cells or follicles of the thymus, but they are completely closed, and do not communicate with any central cavity. They are filled by fluid and cytoblasts, which are the analogues of those in the cells of the thymus; and are held together by fibro-cellular tissue. The cytoblasts are not materially different from those of the thymus; in the young animal they lie close together on the inner surface of the containing cell, like, Oesterlen says, "a tesselated epithelium;" but from this position they detach themselves and float freely in the fluid of the cavity. That they have the relation of nuclei is proved, according to Mr. Simon, by their being not unfrequently found as the nuclei of cells, about $\frac{1}{1100}$ of an inch in diameter. He shows also that a thyroid, or an organ representing it, exists in all vertebrate animals, appearing to have relation to the development of their nervous centres, always maintaining an intimate relation to the vascular supply of the brain, existing in certain fishes as a mere diverticulum to the cerebral circulation, and in the animals above them having a super-addition of glandular structure.

*Spleen.* Oesterlen and Mr. Simon agree that the Malpighian bodies of the spleen are not vesicles, but aggregations of cytoblasts (analogous to those of the vascular glands already described) which are herein collected in small bodies; but these have no inclosing cell wall; each lies within a kind of capsule of capillary vessels, receiving themselves no vessels into their interior. They are held together by an amorphous transparent substance with obscure fibres; among which Oesterlen believes he once detected lymphatic vessels. He has not always found the Malpighian bodies in any of the animals in which he has examined them. Most of the corpuscles or cytoblasts of the spleen, whether scattered through its red substance, or collected in the Malpighian bodies, appear like those of the thymus, but they vary in their contents; others are larger and are merely close-pressed aggregates of granules; others are partially surrounded by darker granules; and others, in different species, differ from all these, approaching the characters of blood-corpuscles.

*Renal Capsules.* Oesterlen has described the structure of these more minutely than that of any other vascular gland. He finds inconstancy in the distinction, proportions, and colours of the cortical and medullary substances. His account of the blood-vessels accords with Müller's. He finds no central cavity (except that of the great central vein); but, occasionally, hollow, elongated spaces of conical shape, which have no lining membrane, and are empty, or contain a thick grayish-white fluid. The appearance of radiating striæ in the cortical substance of these organs is due to groups of the small corpuscles or cytoblasts of which these, like the other vascular glands, are chiefly composed; and of which, in these, many are grouped together with fat-cells and molecules in the form of nearly parallel cylinders or elongated cones; each group being, as Mr. Simon has discovered, inclosed in a tube of very delicate membrane. The medullary substance of the renal capsules, and that which intervenes between the tubes full of corpuscles consist, according to Oesterlen, of cytoblasts, uniformly scattered, and with these are mingled minute molecules, and small collections of fat-cells or particles.

The various microscopic objects found in the renal capsules are, according to both Oesterlen and Simon—(*a*) minute oil or fat particles, either scattered or grouped in round, oval and retort-shaped flattened masses; (*b*) similar groups of fat particles collected round the proper cytoblasts of the organs, but devoid of separate cell-walls; (*c*) the cytoblasts, pale, roundish or oval, disc-like, or more commonly, concavo-convex: some have but the appearance of a nucleolus; in others it is distinct, central, or scattered, or marginal; (*d*) similar cytoblasts surrounded by oval cells; (*e*) four other forms are enumerated by Oesterlen, which are probably only varieties of the preceding.

The preceding statements justify the conclusion drawn by both the authors that these four organs are so similar, that they may be classed as members of one order. To say nothing of their likeness in obvious characters, the essential constituents of all, their peculiar corpuscles, are similar in all, though admitting of distinction in their best marked states. In all, these corpuscles have the relation of cytoblasts or nuclei. In all, some of them are collected in groups of definite, though various form; which groups are in all except the spleen, inclosed in vesicles or tubes formed by delicate membrane, (primary, limitary, or basement membrane), the exterior of which is covered by capillary blood-vessels.

Oesterlen, who cites, besides these analogies, many others of less importance, includes, in the same class with these four organs, the lymphatic glands, the pineal and pituitary glands, the choroidal gland in the eye of fish, and the greenish gland on each side of the gastric sac in crabs, and suspects that many structures in invertebrata will be brought into the same class. In the lymphatic glands he finds, besides those of the lymph, a variety of corpuscles like those in the renal capsules. They may be generally distinguished into two kinds; viz., fat cells and molecules, as in the renal capsules, and the cytoblasts [which, doubtless, are the same as those composing the layer of granular matter described by Mr. Goodsir]. These cytoblasts are various, but the majority cannot be distinguished in any way from the cytoblasts of the thymus. Moreover, Oesterlen says that he has found, in an inguinal gland of a fœtal calf, gland-vesicles like those of the thymus, parotid, &c., formed by transparent membrane, with traces of capillary network on it, and full of cytoblasts, with which fat-molecules and fat-cells are mingled.

Oesterlen describes a very similar structure in the pituitary gland: and he says the pineal gland is similarly composed.

But to know the number of organs which may be included in this class or family, is less important than to determine to what other class of organs they are in nearest relation. It is clear that (as many have believed, but none have proved), they are, in all essential characters, *glands*, and that the name of either vascular glands, or glands without ducts, is appropriate to them. Their chief analogies to the true glands are seen in (*a*) the constant possession of cytoblasts, or nuclei, analogous to the nuclei of the true gland-cells; for even in the latter it is probable that the nuclei, rather than the cells, are the most active and essential apparatus; (*b*) the general existence of the structureless (limitary) membrane inclosing the cytoblasts, as the membrana propria of the true glands incloses the secreting cells (the spleen in which it is absent having perhaps its analogue in the liver among the true glands); (*c*) the arrangement

LIBRARY OF THE

Orleans Parish Medical Society of the capillary vessels on the exterior of the vesicles or other collections of cytoblasts in the glands without, just as in those with, ducts. Thus, the glands without ducts possess all the apparatus which, in the true glands, is provided for secretion. They differ from them in that the formation of cells around their cytoblasts is exceptional, and that their secretion is poured into closed cavities, not into open canals.

Assuming then, that the common occupation of these organs consists in withdrawing from the blood some material which they may (probably after some elaboration) discharge into it again,—the next question is,—What is more particularly the function of each of these glands? In answer only one thing is proved; Mr. Simon's observations prove that in the hybernating animals the thymus forms in itself a store of fat, to be consumed in the maintenance of the temperature during hybernation; and this is more than ever yet was proved of any of these glands. His and former observations also render it highly probable that in other than the hybernant animals, the thymus, during all its temporary existence, is occupied in sequestrating some material from the blood to be restored to it again, in the same or (more probably) some other form. He believes that this material is always such as may be consumed in the service of respiration, "the thymus gland fulfilling its use as a sinking fund in the service of respiration." [But the evidence appears to me insufficient for this conclusion, or even opposed to it; for when in hybernation the gland performs this function, and performs it in the highest degree, it is temporarily adapted for it, not by temporary development, but, as all the analogies of the formation of fat in other cases show, by temporary degeneration. The formation of an extraordinary quantity of fat in any part expresses a defective nutrition of that part; and when the thymus of hybernants accumulates fat at the approach of their winter sleep, it is probably rather because some process in general nutrition to which it before ministered is ceasing, than because it is now about to discharge in an extraordinary degree its ordinary function. I should regard the fatty degeneration or fatty atrophy of the thymus at the approach of each winter-sleep as an annual recurrence of a process analogous to that atrophy by diminution or total removal of substance which takes place once for all in the animals in which the thymus is not persistent. In each case the atrophy is an indication that the necessity for the ordinary acts of the thymus has ceased; but in the hybernants it is for new circumstances made to minister to a new purpose, till, at the cessation of the winter-sleep, and the recommencement of new growth, it begins again to be truly developed, and to form the more highly organic azotized compounds which it may restore to the blood for the nutrition of the fresh-growing tissues.]

For the thyroid gland, Mr. Simon believes (chiefly on the evidence of its comparative anatomy), that it supplies, in its simplest state, a vascular diverticulum to the stream of the cerebral circulation, and that in its higher development, its secretion bears some essential relation to the nutrition of the brain, such that, for instance, while the brain is at rest it may be separating from the blood the same materials as the brain in action takes from the blood.

In like manner he holds the spleen to be as a diverticulum to the systematic circulation when the vessels are filled after taking food; and, by the secretion of the Malpighian corpuscles, an organ in which nutritive matter may be stored up till the system needs it. And, lastly, he thinks the renal capsules may have with the generative system some such relation in alternating secretion as he supposes the thyroid gland to have with the brain.

In the place of these several theories, Oesterlen, in a long discussion, enunciates but one, and that not a new one; namely, that the acts of the glands without ducts are the taking of fluid from the blood, from which as a cytoblastema their cytoblasts are formed; and that these, after their completed development, liquefy and restore to the blood a material more fitted for nutrition than that which it gave for them. [And indefinite and incomplete as this theory is, I must confess it appears to me to express all that can as yet be considered very probable in the general physiology of the glands without ducts.]

